# Vancomycin Enemas as Adjunctive Therapy for Clostridium difficile Infection

**DOI:** 10.14740/jocmr2117w

**Published:** 2015-04-08

**Authors:** Mark Malamood, Eric Nellis, Adam C. Ehrlich, Frank K. Friedenberg

**Affiliations:** a Gastroenterology Section, Temple University School of Medicine, Philadelphia, PA, USA

**Keywords:** *C. difficile* infection, Colectomy, Enema, Vancomycin

## Abstract

**Background:**

For severe, complicated *Clostridium difficile* infection (CDI), concomitant treatment with IV metronidazole and oral vancomycin is usually prescribed. Sometimes vancomycin per rectum (VPR) is added to increase colonic drug delivery. Our purpose was to examine clinical outcomes of patients with CDI treated with VPR and compare results to a matched control group.

**Methods:**

This was a retrospective case-control study in a setting of tertiary-care ICU on diarrhea patients with a positive toxin test for *C. difficile*. We identified all ICU patients prescribed VPR from January 2003 to December 2013. The dose of VPR mixed in 100 cc of tap water ranged from 125 to 250 mg Q 6 - 8 hours. All patients had diarrhea and a positive test for *C. difficile* toxin. Included patients received ≥ 4 doses of VPR. The primary outcome was the combined endpoint of colon surgery or death. We matched VPR cases 1:2 with CDI controls that had identical APACHE II scores.

**Results:**

We identified 24 CDI patients who received VPR and met inclusion criteria: 11 male, mean age 61.8 ± 15.9 years. All patients received concomitant CDI therapy. Four patients (16.7%) required colectomy, and overall mortality was 45.8%. For the 48 controls, need for surgery was identical (16.7%; P = 1.00). The mortality rate also did not differ (41.7%; P = 0.74). For the combined outcome of surgery or death, the rate was 45.8% for the controls and 50.0% for the VPR group (P = 0.73).

**Conclusion:**

In a case-control study, the use of VPR was not demonstrated to reduce the need for colectomy or decrease mortality. Based on our modest sample size and failure to show efficacy, we cannot strongly advocate for the use of VPR.

## Introduction

Despite widespread awareness among healthcare workers of the risk factors for *Clostridium difficile* infection (CDI), there have been large increases in both its incidence and severity over the last 20 years [1-6]. This has translated into higher patient morbidity and mortality as well as an increased economic burden on our health care system [7]. Much of the blame for this trend has been attributed to a particularly virulent strain of *C. difficile*, BI/NAP1 or ribotype 27, which produces elevated levels of toxin and has led to numerous outbreaks of severe CDI [1, 6, 8, 9]. Troublingly, the increase in cases of severe CDI has been coupled with a rise in the rate of colectomy and death for infected patients [9]. For the treatment of severe CDI, defined as a leukocytosis ≥ 15,000 or rise in creatinine ≥ 50% above the pre-morbid level, oral vancomycin is recommended as first-line therapy rather than oral metronidazole [10]. Oral metronidazole is rapidly (0.25 - 1.0 h) and efficiently (≥ 90%) absorbed, but only 6-15% of the original dose reaches the inflamed colon via colonic secretion [11]. As CDI is treated and colitis resolves, the average stool concentration of metronidazole decreases from 9.3 to 1.2 µg/g, below the MIC for *C. difficile* which ranges from 2 to 32 µg/g [11-14]. Comparatively, oral vancomycin is poorly absorbed and colonic vancomycin levels after oral administration reach 64 - 880 µg/g [15]. This concentration is well above the MIC of 1.0 - 2.0 µg/mL for *C. difficile* [12-16]. Concurrent intravenous metronidazole may be added for those with severe disease complicated by hypotension, ileus, or megacolon, otherwise known as severe, complicated CDI [17-19]. In these situations, some institutions also use add-on therapy with vancomycin per rectum (VPR) due to concern about colonic delivery of oral drugs. This option is endorsed by both the Infectious Disease Society of America and the European Society for Microbiology and Infectious Disease [18, 19]. However, the evidence for VPR is limited as most studies reported to date have been case reports [20-22]. In the only three case series to date, VPR was found to be effective in decreasing the need for colectomy and the rate of death [23, 24]. The aim of our study was to expand upon the currently available evidence for the use of VPR by analyzing outcomes at our institution. Our study is the first to use a carefully selected control group for comparison.

## Patients and Methods

We first obtained IRB approval to review clinical information. Using pharmacy records, we identified all inpatients at our institution prescribed VPR from January 2003 to December 2013. Charts were manually reviewed. Included patients had diarrhea and a positive stool test for *C. difficile* toxin by EIA (Wampole *C. difficile* Tox A/B II, Wampole Laboratories, Princeton, NJ) and/or pseudomembranes identified on colonoscopy or sigmoidoscopy. We restricted our definition of a study case to patients who were in the ICU at the initiation of treatment and received ≥ 4 doses (i.e. ≥ 1 day) of VPR. APACHE II scores for the day of CDI diagnosis were calculated for all patients meeting inclusion/exclusion criteria. Selected patients could not have an alternative cause for symptoms. Information was recorded for the entire hospitalization until discharge. All patients received intravenous fluids and electrolyte replacement as determined by their provider but we did not systematically record other conservative measures such as the use of anti-diarrheals. The primary combined outcome variable of interest was colectomy or death. We selected our controls from a list of ICU patients diagnosed with CDI in our hospital over the same period of time, excluding those prescribed VPR. The list from our medical records department was scrambled alphabetically prior to chart review. Control patient charts were manually reviewed and APACHE II scores were calculated for their day of CDI diagnosis. For each case patient, two control patients in the ICU with matching year of CDI diagnosis and APACHE II score were selected. After identifying sufficient controls, we stopped reviewing charts. The most common reasons that patients from the control list were excluded were insufficient data to calculate APACHE II score, lack of required CDI diagnostic criteria, and being a control that had already matched to a patient.

### Delivery of vancomycin by enema

The dose of VPR for all patients was 125 or 250 mg Q 6 h. Vancomycin Hydrochloride for Injection USP (Hospira Inc., Lake Forest, IL) was mixed thoroughly with 100 cc of tap water prior to instillation. Drug delivery was most commonly performed using a rectal tube with the patient in the supine or left lateral decubitus position. The tube was clamped after delivery usually for a minimum of 30 min to allow adequate mucosal contact time.

### Statistical analysis

Prior to inferential statistics, we assessed the distribution of continuous data. For parametrically distributed continuous data, we performed Student’s *t*-test to compare data between groups. Data were presented as means ± standard deviation. For non-parametric comparisons, the Wilcoxon signed-rank test was utilized. Non-parametric data were presented as median with interquartile range (IQR). For comparison of categorical data, 2 × 2 tables were constructed and significance was calculated using the Chi-square test. In order to determine independent predictors of mortality we developed a logistic regression model. The dependent variable was the combined endpoint of colectomy/death. *A priori*, treatment with vancomycin enema was selected as one of the independent explanatory variables. All calculated P-values were two-tailed, with significance set at P ≤ 0.05. SPSS version 22 (IBM Corporation, Armonk, NY) was used for calculations. No formal power or sample size calculation was performed due to the limited number of cases available for analysis.

## Results

We identified 43 CDI patients in the ICU who received VPR during the study period. We excluded 18 patients because they did not receive ≥ 4 doses of VPR and one patient who received VPR after colonic surgery. Therefore we included 24 CDI patients. A total of 637 charts were reviewed in order to find 48 matching control CDI patients. Table 1 compares the characteristics of the cases and controls. The patients were primarily middle-aged to elderly with profound hypoalbuminemia. The median length of stay was nearly a month with roughly half of the days as a resident of the intensive care unit. The mean APACHE II scores (a matched variable) was 20 for both groups predicting a 40% in-hospital mortality [25]. Over 90% of patients in both groups had severe or severe-complicated CDI.

**Table 1:** Characteristics of Study Patients and Controls

	All	Rectal vancomycin (n = 24)	No rectal vancomycin (n = 48)	P value
Age (SD), years	61.3 (15.4)	61.8 (15.9)	61.1 (15.3)	0.86
Gender (% male)	36 (50)	11 (45.8)	25 (52.1)	0.62
Immunosuppression* (%)	12 (16.7)	4 (17.4)	8 (16.7)	0.94
Albumin (SD), g/dL	1.95 (0.63)	2.01 (0.59)	1.91 (0.66)	0.56
APACHE II (SD)	20.0 (5.4)	20.0 (5.4)	20.0 (5.5)	1.00
Overall LOS (median, IQR)	26.5 (16.5 - 46.5)	25.0 (19.5 - 37.5)	28.0 (15.5 - 48.0)	0.62
ICU LOS (median, IQR)	13.0 (2.5 - 25.0)	12.0 (0 - 27.5)	14.0 (3.5 - 23.5)	0.72
Episode severity				0.18
Mild-moderate	4 (5.6)	1 (4.2)	3 (6.3)	
Severe	28 (38.9)	6 (25)	22 (45.8)	
Severe complicated	40 (55.6)	17 (70.8)	23 (47.9)	

LOS: length of stay. *Includes prednisone ≥ 20 mg, antineoplastic, antimetabolite, and any immune modulator therapy.

**Figure 1:**
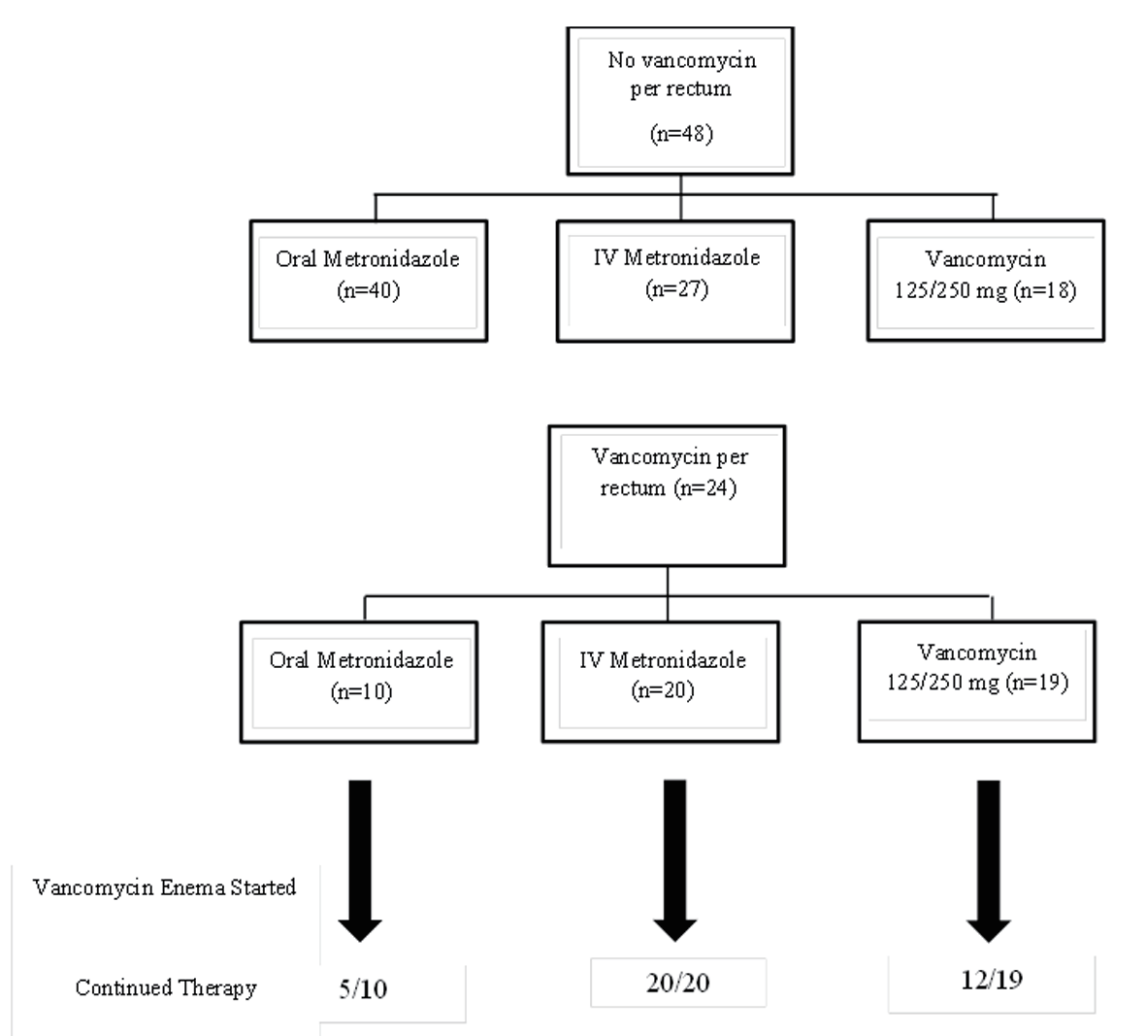
CDI treatment for the control group (top) and VPR group (bottom). To be listed as exposed to antimicrobial, the patient had to receive at least 1 day of that therapy. Number of antibiotics > number of patients due to concomitant therapy.

Figure 1 highlights therapy for CDI. The numbers for each therapy indicates that the patient received at least 1 day of that therapy. The ratio of drug exposure/patient is greater than 1 because therapy switches were common. All patients in both groups at some point received either oral or IV metronidazole. However, only 37.5% of controls received oral vancomycin, while 79.2% of VPR cases received this therapy (P = 0.001). For both cases and controls, many patients were on more than one CDI therapy simultaneously. As can be seen for the lower half of the figure, continuation of baseline therapy was common after adding on VPR. If a patient was receiving IV metronidazole and/or oral vancomycin, this therapy was not stopped. For many, VPR represented their third concomitant CDI therapy.

**Figure 2:**
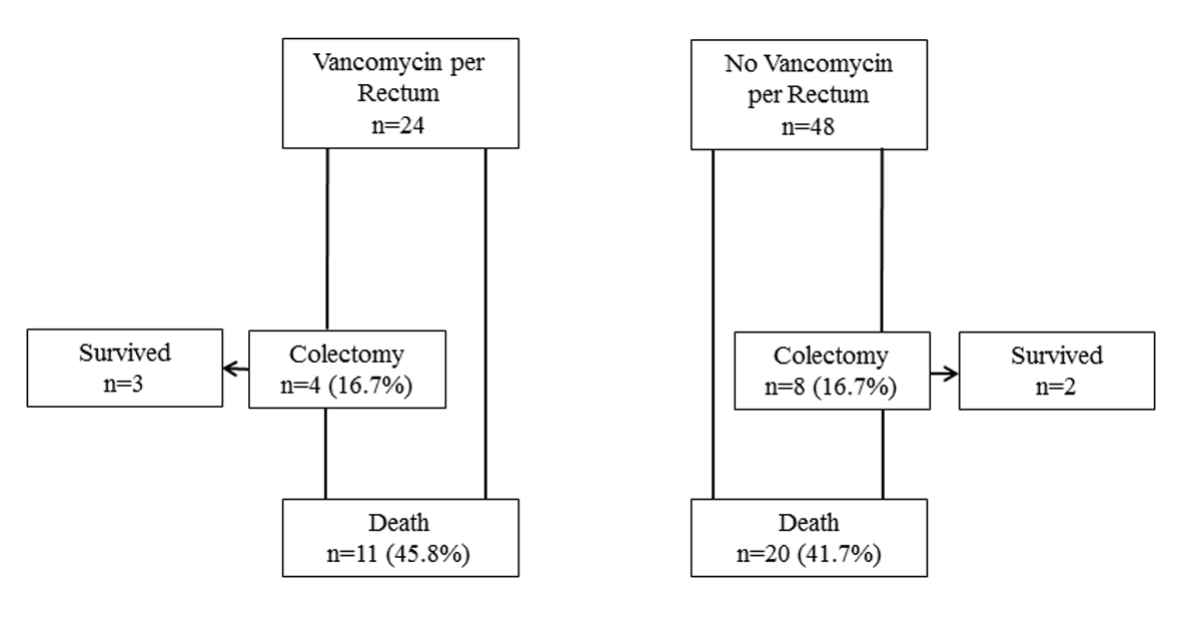
Flow chart for combined primary outcomes.

For the major outcomes, surgery was performed in eight (16.7%) control patients not receiving VPR, and four (16.7%) VPR patients (P = 1.00) (Fig. 2). Death occurred in 20 (41.7%) control patients and 11 (45.5) VPR patients (P = 0.74). In the control group, six patients died after surgery compared to three in the VPR group. Therefore, for the combined outcome of surgery or death, the rate was 45.8% for the controls and 50.0% for the VPR group (P = 0.73). For the VPR group, we explored the timing of starting VPR on outcomes. The mean number of days of CDI treatment before starting VPR was identical for the patients with (n = 12) and without (n = 12) a favorable outcome (10.1 ± 15.7 vs. 10.1 ± 8.7, P = 1.00). The combined outcome of surgery or death for those started on VPR within 7 days of CDI treatment was insignificantly lower (five vs. seven patients) than for those started at a later time (P = 0.09).

**Figure 3:**
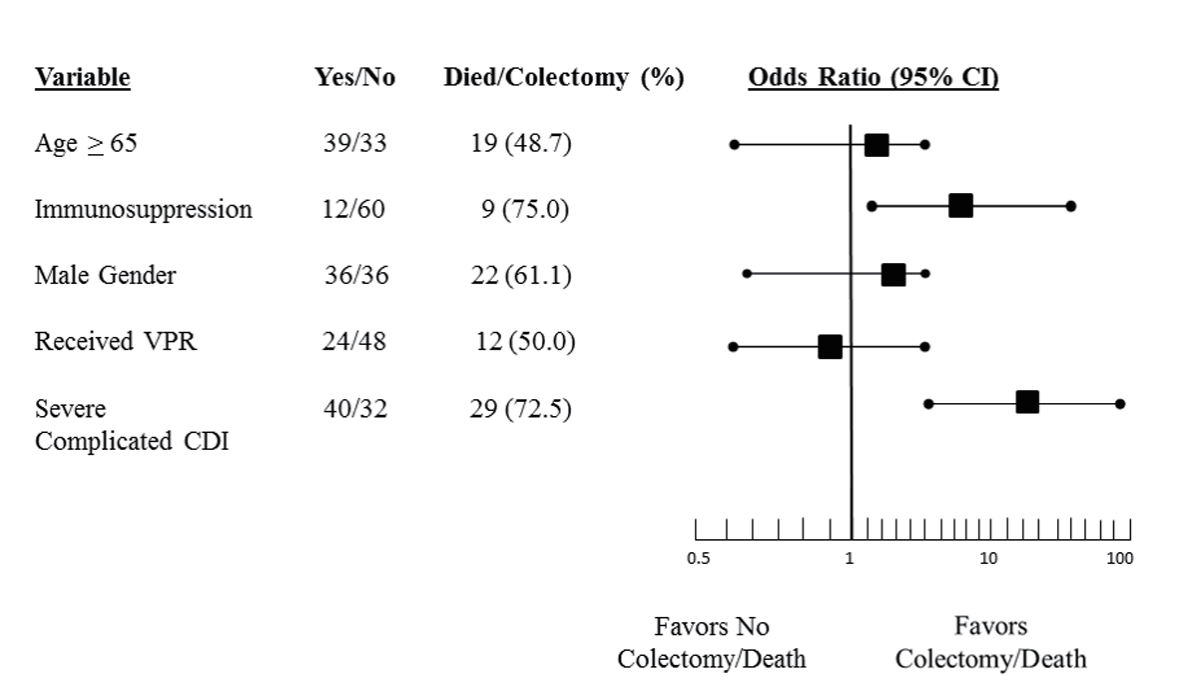
Plot of odds ratio and 95% confidence interval of variables independently associated with the combined endpoint of colectomy and death.

We performed a logistic regression analysis to estimate the effect of VPR on the occurrence of death or surgery. The results are presented in Figure 3. We found that underlying immunosuppression (OR: 8.7, 95% CI: 1.3 - 57.1) and the presence of severe complicated disease (OR: 19.0, 95% CI: 4.3 - 84.4) were independently associated with this combined outcome. Use of VPR was not associated with outcome (OR: 0.6, 95% CI: 0.2 - 2.2).

## Discussion

It has been established that the preferred treatment for severe CDI is oral vancomycin [10, 17-19]. In patients with severe CDI and certain complications, namely hypotension, toxic megacolon, and colonic ileus, adjunctive therapy with intravenous metronidazole may be beneficial. Despite this, many patients develop progressive disease leading to colonic perforation, peritonitis, and septic shock. Under these circumstances mortality is very high, and colectomy may be lifesaving [26-29]. Our main aim was to evaluate the efficacy of VPR as an additional adjunctive treatment for severe and severe complicated CDI primarily as a means to prevent colectomy and/or death. Our study is the first case-control study comparing VPR therapy with oral and/or IV CDI therapy alone. After matching ICU patients who did and did not receive VPR in a 1:2 ratio based on APACHE II score at time of CDI diagnosis, we found that VPR had no statistically significant effect on preventing colectomy and/or death. The number of patients who required colectomy, died, or had the combined endpoint of colectomy/death, did not differ between the VPR+ and VPR- groups (all P > 0.05). The timing of VPR (how quickly it was added on after treatment initiation) seemed to make no difference.

VPR is utilized in order to augment colonic drug levels supplementing oral delivery [18, 19]. In theory, VPR can successfully deliver drug to the distal of one-third of the colon. This is supported by a study of mesalamine for ulcerative colitis whereby rectal delivery of 100 mL of medication reached as high as the descending colon [30]. There have been a few case reports and small series commenting on the efficacy of VPR in severe CDI, with and without complications [20-24]. These studies have reported predominantly positive results (Table 2). The doses of VPR used have been higher than in our study, typically 1 g of vancomycin solution mixed in 500 mL 0.9% normal saline Q 6 h. As in our study, there was frequent use of concomitant oral vancomycin and intravenous metronidazole. Based on the available literature, the Infectious Diseases Society of America has assigned a grade of C-III for the use of adjunctive therapies including IV metronidazole and VPR in severe complicated CDI, indicating general recommendations cannot be strongly endorsed [18].

**Table 2:** Published Reports of VPR

Author (year)	Case report or series (n)	Dose of VPR	Concomitant therapies	Comments
Pasic et al [20] (1993)	Report	2 g loading with 100 mg q6h and 100 mg PRN watery stool	No	Colitis resolution; death from sepsis on POD 52
Shetler et al [21] (2001)	Series (7)	250 mL of premixed solution (1 g vancomycin in 1 L sterile H_2_O) q6h	Yes (all IV metronidazole, 5/7 oral vancomycin)	Colitis resolution in four of the seven patients
Nathanson et al [22] (2001)	Report	500 mg in 500 mL saline BID	Yes (IV metronidazole for 10/10 days)	Colitis resolution with discharge to home
Apisarnthanarak et al [23] (2002)	Series (9)	Variable (0.5 - 1 g q4 - q12h)	Yes (all IV/oral metronidazole, 7/9 oral vancomycin)	Resolution in 89%
Kim et al [24] (2013)	Series (47)	1 g q6h	Yes (oral vancomycin and IV)	Resolution without surgery in 70%

The main strength of our study is the inclusion of a well-matched control arm. We studied a relatively homogeneous group of patients, those requiring ICU care, who would be reasonable candidates for VPR. We elected to match overall disease severity between groups using a well-validated scoring system, the APACHE II [25]. In fact the predicted and actual mortality rate for our study group was remarkably similar (APACHE II 40% vs. actual 45.8%). As Table 1 shows, there were no differences in any other measured variables between the two arms of the study that approached statistical significance. Matching on several known confounders hopefully led to matching on important unmeasured variables. A final important strength of our study is the use of well-defined outcomes, surgery and death, eliminating the possibility of outcome misclassification.

We acknowledge several weaknesses within our study as well. First, our sample size is small and it is possible our results represent a type II error. We attempted to reduce error margins using a case to control ratio of 1:2 but a larger sample of cases would improve our point estimates and strengthen the confidence of our findings. Secondly, our study is observational and we were unable to control for the antibiotic regimen used to treat our patients. As shown in Figure 1, patients in both arms of the study received varying regimens consisting of vancomycin oral, metronidazole oral, and/or metronidazole IV. A higher exposure to oral vancomycin was seen in the VPR group. We also could not control for the timing of VPR as some patients received this therapy within days of diagnosis and others after a week or more. Finally, our hospital has just begun to report the strain of *C. difficile*, but this was not available for the study period.

Independent risk factors for the endpoint of colectomy/death in our study were the use of immunosuppression and having severe complicated disease as opposed to mild-moderate or uncomplicated severe disease. Of the 40 patients from both groups with severe complicated disease, 29 (72.5%) died or had a colectomy. All patients who died experienced hypotension/shock at some point fulfilling the criteria for severe complicated disease. Overall, 12 (30%) had radiographic evidence of an ileus and/or toxic megacolon. Our mortality rate is much higher than other studies which have reported this incidence; however, we studied a very ill subset of individuals [31]. Moreover, our reported mortality rate is all-cause as it is not possible to ascertain the incidence of CDI-attributable cases due to the retrospective nature of our study.

In conclusion, our study is the first case-control series to investigate the use of VPR for the treatment of severe CDI. In contrast to previously reported case reports and case series, we found that VPR did not decrease the need for colectomy and/or death when compared to standard therapy. In light of this study’s limitations, we cannot advocate for or against the use of VPR to treat severe CDI. Additional, larger case-control studies, or preferably controlled trials, are needed to further investigate this therapy.

## Statement of Interests

None of the three authors have any personal or funding interests to declare.

## Abbreviations

CDI: *C. difficile* infection
LOS: length of stay
VPR: vancomycin per rectum

## References

[1] Bartlett JG (2006). Narrative review: the new epidemic of Clostridium difficile-associated enteric disease. Ann Intern Med.

[2] To KB, Napolitano LM (2014). Clostridium difficile infection: update on diagnosis, epidemiology, and treatment strategies. Surg Infect (Larchmt).

[3] Ricciardi R, Rothenberger DA, Madoff RD, Baxter NN (2007). Increasing prevalence and severity of Clostridium difficile colitis in hospitalized patients in the United States. Arch Surg.

[4] Jarvis WR, Schlosser J, Jarvis AA, Chinn RY (2009). National point prevalence of Clostridium difficile in US health care facility inpatients, 2008. Am J Infect Control.

[5] McDonald LC, Owings M, Jernigan DB (2006). Clostridium difficile infection in patients discharged from US short-stay hospitals, 1996-2003. Emerg Infect Dis.

[6] Pepin J, Valiquette L, Alary ME, Villemure P, Pelletier A, Forget K, Pepin K (2004). Clostridium difficile-associated diarrhea in a region of Quebec from 1991 to 2003: a changing pattern of disease severity. CMAJ.

[7] Dubberke ER, Wertheimer AI (2009). Review of current literature on the economic burden of Clostridium difficile infection. Infect Control Hosp Epidemiol.

[8] Miller M, Gravel D, Mulvey M, Taylor G, Boyd D, Simor A, Gardam M (2010). Health care-associated Clostridium difficile infection in Canada: patient age and infecting strain type are highly predictive of severe outcome and mortality. Clin Infect Dis.

[9] Miller MA (2007). Clinical management of Clostridium difficile-associated disease. Clin Infect Dis.

[10] Zar FA, Bakkanagari SR, Moorthi KM, Davis MB (2007). A comparison of vancomycin and metronidazole for the treatment of Clostridium difficile-associated diarrhea, stratified by disease severity. Clin Infect Dis.

[11] Bolton RP, Culshaw MA (1986). Faecal metronidazole concentrations during oral and intravenous therapy for antibiotic associated colitis due to Clostridium difficile. Gut.

[12] Wong SS, Woo PC, Luk WK, Yuen KY (1999). Susceptibility testing of Clostridium difficile against metronidazole and vancomycin by disk diffusion and Etest. Diagn Microbiol Infect Dis.

[13] Freeman J, Stott J, Baines SD, Fawley WN, Wilcox MH (2005). Surveillance for resistance to metronidazole and vancomycin in genotypically distinct and UK epidemic Clostridium difficile isolates in a large teaching hospital. J Antimicrob Chemother.

[14] Aspevall O, Lundberg A, Burman LG, Akerlund T, Svenungsson B (2006). Antimicrobial susceptibility pattern of Clostridium difficile and its relation to PCR ribotypes in a Swedish university hospital. Antimicrob Agents Chemother.

[15] Keighley MR, Burdon DW, Arabi Y, Williams JA, Thompson H, Youngs D, Johnson M (1978). Randomised controlled trial of vancomycin for pseudomembranous colitis and postoperative diarrhoea. Br Med J.

[16] Bishara J, Bloch Y, Garty M, Behor J, Samra Z (2006). Antimicrobial resistance of Clostridium difficile isolates in a tertiary medical center, Israel. Diagn Microbiol Infect Dis.

[17] Surawicz CM, Brandt LJ, Binion DG, Ananthakrishnan AN, Curry SR, Gilligan PH, McFarland LV (2013). Guidelines for diagnosis, treatment, and prevention of Clostridium difficile infections. Am J Gastroenterol.

[18] Cohen SH, Gerding DN, Johnson S, Kelly CP, Loo VG, McDonald LC, Pepin J (2010). Clinical practice guidelines for Clostridium difficile infection in adults: 2010 update by the society for healthcare epidemiology of America (SHEA) and the infectious diseases society of America (IDSA). Infect Control Hosp Epidemiol.

[19] Debast SB, Bauer MP, Kuijper EJ (2014). European Society of Clinical Microbiology and Infectious Diseases: update of the treatment guidance document for Clostridium difficile infection. Clin Microbiol Infect.

[20] Pasic M, Jost R, Carrel T, Von Segesser L, Turina M (1993). Intracolonic vancomycin for pseudomembranous colitis. N Engl J Med.

[21] Shetler K, Nieuwenhuis R, Wren SM, Triadafilopoulos G (2001). Decompressive colonoscopy with intracolonic vancomycin administration for the treatment of severe pseudomembranous colitis. Surg Endosc.

[22] Nathanson DR, Sheahan M, Chao L, Wallack MK (2001). Intracolonic use of vancomycin for treatment of clostridium difficile colitis in a patient with a diverted colon: report of a case. Dis Colon Rectum.

[23] Apisarnthanarak A, Razavi B, Mundy LM (2002). Adjunctive intracolonic vancomycin for severe Clostridium difficile colitis: case series and review of the literature. Clin Infect Dis.

[24] Kim PK, Huh HC, Cohen HW, Feinberg EJ, Ahmad S, Coyle C, Teperman S (2013). Intracolonic vancomycin for severe Clostridium difficile colitis. Surg Infect (Larchmt).

[25] Knaus WA, Draper EA, Wagner DP, Zimmerman JE (1985). APACHE II: a severity of disease classification system. Crit Care Med.

[26] Lamontagne F, Labbe AC, Haeck O, Lesur O, Lalancette M, Patino C, Leblanc M (2007). Impact of emergency colectomy on survival of patients with fulminant Clostridium difficile colitis during an epidemic caused by a hypervirulent strain. Ann Surg.

[27] Jaber MR, Olafsson S, Fung WL, Reeves ME (2008). Clinical review of the management of fulminant clostridium difficile infection. Am J Gastroenterol.

[28] Longo WE, Mazuski JE, Virgo KS, Lee P, Bahadursingh AN, Johnson FE (2004). Outcome after colectomy for Clostridium difficile colitis. Dis Colon Rectum.

[29] Sailhamer EA, Carson K, Chang Y, Zacharias N, Spaniolas K, Tabbara M, Alam HB (2009). Fulminant Clostridium difficile colitis: patterns of care and predictors of mortality. Arch Surg.

[30] van Bodegraven AA, Boer RO, Lourens J, Tuynman HA, Sindram JW (1996). Distribution of mesalazine enemas in active and quiescent ulcerative colitis. Aliment Pharmacol Ther.

[31] Loo VG, Poirier L, Miller MA, Oughton M, Libman MD, Michaud S, Bourgault AM (2005). A predominantly clonal multi-institutional outbreak of Clostridium difficile-associated diarrhea with high morbidity and mortality. N Engl J Med.

